# Household dietary diversity and Animal Source Food consumption in Ethiopia: evidence from the 2011 Welfare Monitoring Survey

**DOI:** 10.1186/s12889-016-3861-8

**Published:** 2016-11-25

**Authors:** Abdulhalik Workicho, Tefera Belachew, Garumma Tolu Feyissa, Beyene Wondafrash, Carl Lachat, Roosmarijn Verstraeten, Patrick Kolsteren

**Affiliations:** 1 Department of Epidemiology, Jimma University College of Health Sciences, Jimma, Ethiopia; 2 Department of Population and Family Health, Jimma University College of Health Sciences, Jimma, Ethiopia; 3 Department of Health Education and Behavioral Sciences, Jimma University College of Health Sciences, Jimma, Ethiopia; 4 Nutrition and Child Health Unit, Department of Public Health, Institute of Tropical Medicine, Nationalestraat 155, Antwerp, 2000 Belgium; 5 Department of Food Safety and Food Quality, Ghent University, Coupure links 653, Ghent, B 9000 Belgium

**Keywords:** Household dietary diversity, Animal Source Food consumption, Ethiopia

## Abstract

**Background:**

It is imperative to track dietary quality and progress in nutritional outcomes in a population to develop timely interventions. Dietary diversity is a commonly used proxy to assess dietary quality in low-income countries. This study identified predictors of household dietary diversity in Ethiopia and pattern of consumption of animal source food (ASF) among households.

**Methods:**

Secondary data were analyzed from the 2011 Ethiopian Welfare Monitoring Survey (WMS). This survey used a structured questionnaire to collect socio-demographic and economic data. Dietary data were collected using a dietary diversity questionnaire measuring dietary diversity over the past 1 week. A Household Dietary Diversity Score (HDDS) was constructed according to the Food and Agricultural Organization (FAO) guidelines. Consumption of ASFs is described by its distribution among the regions and by HDDS. Multiple logistic regression analysis was fitted to identify independent predictors for HDDS.

**Results:**

A total of 27,995 households were included in the analyses. A little over half of the study households (52.2%) had more than four household members, and 75% of households were male headed. The mean HHDS was five food groups. Cereals were the most commonly (96%) consumed food groups. Fish, egg and fruits, on the other hand, were the least consumed food groups. ASFs were consumed in greater proportion among households with higher HDDS. Being part of the higher and middle socio economic strata (*P < 0.001*), literacy (*P < 0.01*), urban residence (*P < 0.01*), male headed household (*P < 0.01*), larger family size (*P <0.01*) and owning livestock (*P < 0.01*) were positively associated with higher HDDS.

**Conclusions:**

Considering these findings, nutrition sensitive interventions which address the problem through economic and educational empowerment and modern technologies supporting agricultural practices need to be designed to increase both local production and increased consumption.

## Background

Optimal nutrition and health is needed to ensure proper development and well-being [1]. It is imperative to track dietary quality and progress of nutritional outcomes in a population to develop timely interventions. Recent developments, including increased attention and funding for nutrition-sensitive interventions, have increased demand for indicators of food consumption and diet quality [2].

Indices of dietary quality are increasingly used to monitor nutritional status and dietary intake of populations [3–5]. In developed countries, these indices are often composed of several dimensions such as nutrient adequacy, dietary diversity, proportionality (more of some food groups and less of others) and moderation (limiting the intake of food constituents that contribute to excess risk) [3]. In low-income countries, where inadequate dietary intake is a key concern, nutrient adequacy alone is often used to indicate dietary quality. However, quantifying intake of nutrients is often expensive, time-consuming and associated with methodological challenges in such settings [6]. Dietary diversity, defined as the number of unique foods or food groups consumed over a given period, has been considered a potential ‘proxy’ indicator to reflect nutrient adequacy [7], and of adequate intake across a range of key micronutrients [8]. Evidence in both high-income and low- and middle-income countries (LMICs) [9, 10] has shown that a higher DDS is associated with an increased nutrient intake and better nutritional status and also household per capita energy consumption [11]. Furthermore, Dietary Diversity Score (DDS) has been identified as a key indicator for surveillance of actions that aim to tackle various nutrition-related problems and food insecurity [12]. Firstly, it provides information on the contribution of different food groups to the diet which sheds light on its quality and nutrient adequacy in a population. Secondly, evidence on how DDS varies across population groups, cultures, and socio-economic level can be obtained and is a key to understand the performance of DDS as an indicator for nutritional surveillance programs.

Consumption of ASF is linked to both dietary diversity and quality [13] and it is one of the main indicators commonly used to assess dietary intake and quality in low and middle income countries [14–16]. Even though there are social, economic and cultural barriers for the intake of ASF [17], its consumption, even in relatively small amounts, is believed to enrich the body with protein and essential micronutrients like iron, zinc, and vitamins [13, 17, 18]. Evidence also indicates that poor physical growth, impaired cognition, morbidity and mortality are strongly associated with poor or inadequate intake of ASF throughout the life cycle [19–23]. Therefore, inclusion of ASFs in the diversification of a diet among the households helps to ensure consumption of quality diet beyond its diversification.

To address nutritional problems, the Ethiopian government has committed itself to develop approaches which consider the multi-sectoral nature of these problems. A recently developed National Nutrition Strategy [24] and National Nutrition Program in the country combined with the government’s engagement in various international initiatives like Scaling Up Nutrition, Feed the Future, and Accelerated Stunting Reduction, demonstrate the government’s effort to reduce malnutrition. Promoting the consumption of a diversified diet is one of the strategies to decrease malnutrition in the revised National Nutrition Program [25]. Therefore, evaluating the dietary diversity and its determinants across the population will inform the various national initiatives and will be used to evaluate interventions. This study examined the determinants of household dietary diversity in Ethiopia. It also assessed food groups consumed across socio-economic score (SES) and their composition at different Household Dietary Diversity Score (HDDS) so that it will give an insight about the quality of food consumed in the households.

## Methods

### Study sample and measurement

Secondary data were analyzed from the 2011 Ethiopian WMS. The survey covered all rural and urban areas of the country, except three pastoral zones of Afar region and six of the Somali region. Different strata were defined for the urban and rural area. Urban areas were classified into three categories based on their population size: major urban centers, medium and small size towns. In the rural area, agro-ecological zones were used as strata. A two-stage stratified cluster design was used to select sample enumeration areas and households from major urban centers and rural areas. For medium and small towns, a three stage stratified cluster design was used to select sample towns, enumeration areas and households. The 2007 Population and Housing Census data was used as a sampling frame. All major urban centers were directly selected. Sample towns (medium & small size) in each region and sample enumeration areas both in urban and rural areas were selected by probability proportional to size based on the number of households from the 2007 Census. All households from all selected enumeration areas were listed. The listing excluded collective quarters like army barracks, hospitals, police camps and boarding schools. Then, a representative sample of 27,995 households was selected from the enumeration areas for the survey. A structured questionnaire was used to collect socio-demographic and economic data. Dietary data were collected using a dietary diversity questionnaire. The questionnaires, translated into local language, were interviewer-administered and were verified for consistency during pretesting. Interviewers were trained before the actual data collection and supervisors kept track of the field procedures and checked the completed questionnaires everyday to ensure the quality of the data. Detailed participant characteristics and data collection methods have been described elsewhere [26]. The study was approved by the Ethical Review Board of Jimma University, College of Health Sciences.

### Household Dietary Diversity Score and Animal Source Food intake

We used a dietary diversity questionnaire containing commonly consumed food items in Ethiopia to calculate the HDDS and evaluate the household dietary practices. The respondents were asked to report the number of days in the past week they consumed categories of the listed food items. The food groups used to construct the HDDS in this study were constructed from the list of food items the participants reported to consume according to the FAO guideline*;* cereals, tubers, legumes, meat, egg, vegetables, fruits, oil, sweets, milk and fish, [27]. Consumers of a food item were defined as follows: consumption of the food item at least once in the past week [28]. Irrespective of the frequency, a household which consumed a food group at least once in a week period was scored as 1.The HDDS was constructed as the sum of numbers of food groups consumed over the past week. Higher score indicates higher diversity, as more food groups were eaten.

In the current sample, the minimum and maximum HDDS were one and eleven respectively. Despite the lack of established cut-offs in terms of number of food groups to indicate adequate or inadequate dietary diversity for HDDS, higher HDDS is desirable and larger number of food groups are required to meet requirements of various nutrients [29]. Therefore, the score is converted into tertiles and the higher tertile of the score was taken as high HDDS and the middle and low tertiles were merged together as low HDDS [30, 31]. In the high HDDS category households consumed seven and more food groups while in the low HDDS less than seven food groups in the previous 1 week.

ASF intake was assessed by counting the consumption of animal source products (meat, milk, egg and fish) by the households over the 1 week period. The consumption of each ASF is then described by its distribution among the regions. Composition of the food groups including ASFs; consumed in the higher and lower categories of HDDS was also examined to assess what additional food groups came in to the plate when a household is in the higher HDDS category which also gives insight about the quality of food consumed in the households.

### Predictors of dietary diversity

The individual characteristics examined as determinants of dietary diversity were age, sex, literacy status of the household head, and illness of any household member over the past 12 months. It was hypothesized that male sex of the household head and being literate were positively associated with dietary quality; while illness in any member of the household was believed to negatively affect the dietary diversity [30–33]. The examined household level factors included residence (urban vs. rural), household size (categorized as 4 and less and above 4 based on the average household size), experience of shock (defined as having experienced at least one of the following circumstances in the past 12 months: drought, crop damage, flood, loss of job, robbery, fire accident or land slide), experience of food shortage in the past 12 months, number of crops cultivated (categorized as 3 and less and above 3 crops based on the average number of crops cultivated) and socio-economic score. As there is no income and expenditure data to measure household’s economic status, SES index was constructed. The index was constructed using principal component analysis based on information on housing conditions, ownership of assets and access to basic service. The items used were, ownership of durable goods (TV, Radio, sofa set, refrigerator), crop land, livestock, house, cash crop production, transportation (car, motor bike, cart, horse, donkey, camel), house construction materials, farming materials, source of drinking water, type of the wall, the roof, ceiling of the house, having toilet, kitchen and electricity. The analysis was performed separately for urban and rural households as the characteristics that define wealth in the two scenarios are expected to differ [32, 33]. Three factors were identified both in urban and rural areas and they explained 63 and 59% of the variations in the variables included in urban and rural areas respectively. Factor loadings having a value > 0.5 were the basis for selecting individual variable for the final factors. The variables included in the final factors were source of drinking water, toilet facility, electricity, type of floor, type of wall, ownership of durable goods (4 items recorded as yes and no ), crop land, livestock, ownership of horses, donkey or cart. For analytical purposes, the score is then categorized into tertiles as low, medium and high SES [32]. The variation in the pattern of consumption of all the food groups across socio-economic score was also tested using the chi square test for trend. HDDS was also described in terms of its distribution in the regions to identify regions with high and low HDDS.

### Statistical analysis

The data were coded and entered in to SPSS for windows [SPSS Inc. version 16.1, Chicago, Illinois]. Descriptive statistical analyses were conducted to determine the characteristics of the households and the types of food groups consumed in the reference period. Mean and standard deviation were reported for continuous variables that are normally distributed and frequency distributions for categorical variables. Bivariate analysis was carried out to test differences in HDDS. Multiple logistic regression analysis was used to identify independent predictors of HDDS after controlling for confounding factors. All covariates showing association with HDDS (*p* < 0.25) were included in the final model. Interaction terms between different predictor variables were evaluated and collinearity was assessed using variance inflation factor. The association between consumption of individual food groups and SES was examined by Chi Square test for trend. Statistical significance was determined by using a *P* < 0.05 as a cut-off point. The results are presented as proportions and the parameters from the model as β coefficients and OR with 95% confidence interval. All the above analysis took into account the survey design by adjusting for sampling weight, strata and clustered nature of the data by using *svy* command on STATA version 13.

## Results

### Characteristics of the study households

A total of 27,995 households were involved in the analysis. A little over half of the study households, 52.2% had more than four household members during the time of the survey. Majority of the respondents were found in the age range of above 50 years (29.9%) followed by 31–40 years (25.9%). All of the respondents were heads of household and 75.0% of them were males. About 40.0% of heads of the households were literate and 21.6% of the study households were from urban residence. Only 7.8 and 26.2% reported to have experienced illness and any kind of shock in the past 12 months. Out of the farming households, as many as 36.9% of the households reported to cultivate more than three types of crops. From the total households, 11.2% of them were categorized as having high socio-economic score (Table 1).

**Table 1:** Characteristics of households

Variable		Frequency (*n* = 27,995)	Percentage (%)
Age of household head	≤20	662	2.4
	21–30	6318	22.6
	31–40	7245	25.9
	41–50	5411	19.3
	>50	8359	29.9
Sex of Household head	Male	20,921	74.7
	Female	7074	25.3
Literacy of household head	Read and write	11,124	39.7
	Don’t read and write	16,871	60.3
Residence	Urban	6037	21.6
	Rural	21,958	78.4
Illness	Yes	2778	7.8
	No	25,817	92.2
Experience of shock in the past 12 months	Yes	7340	26.2
	No	20,655	73.8
Food shortage in the past 12 months	Yes	4047	14.5
	No	23,948	85.5
Household size	≤4	13,375	47.8
	>4	14,620	52.2
Crops cultivated^a^	≤3	7708	63.2
	>3	4498	36.9
Engaged in farming	Yes	21,725	77.6
	No	6270	22.4
Own livestock	Yes	20,970	74.9
	No	7025	25.1
Socio-economic score	Low	17,921	64.0
	Medium	6945	24.8
	High	3129	11.2

^a^ *n* = 12,206 (only those who were engaged in farming practice were asked this question)

### Description of food groups consumed

**Fig. 1:**
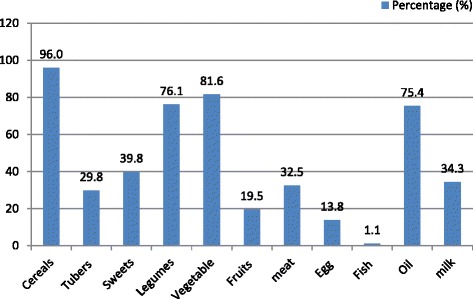
Proportion of food groups consumed by the households

The details of the proportion of households who consumed various types of food groups over the previous week are presented in Fig. 1. The mean HHDS was five food groups. Cereals were the most commonly consumed food group by 96.0% of the households, followed by vegetables (81.6%), legumes (76.1%) and oils (75.4%). Fish, egg and fruits on the other hand were the least consumed food groups, in the study households. The mean HDDS is found to be 5 (±1.9).

### Regional distribution of higher HDDS, farming activity and owning livestock

In all the regions except in the South Nations and Nationalities and People’s Representatives (SNNPR), there was a significant difference between the proportions of households with high and low HDDS. The proportion of households with higher HDDS was highest in Dire Dawa (49.3%), followed by Harari (45.1%) and the capital Addis Ababa (39.0%). Majority of the households in all the regions except Addis Ababa, Diredawa and Afar were also engaged in farming and livestock rearing (Table 2).

**Table 2:** Regional distribution of HDDS, Owning livestock and farming activity^a^

Region	High HDDS		*P*	Engage in farming		*P*	Own livestock		*P*
	n	%		n	%		n	%	
Tigray (*n* = 1816)	389	21	<0.01	1322	73	<0.01	1262	70	<0.01
Afar (*n* = 169)	23	14	<0.01	58	34	<0.01	128	76	0.55
Amhara (*n* = 7278)	988	14	<0.01	5800	80	<0.01	5645	78	<0.01
Oromia (*n* = 10,546)	2418	23	<0.01	8699	83	<0.01	8416	80	<0.01
Somali (*n* = 605)	104	17	<0.05	403	67	<0.01	493	81	<0.01
Benishangul (*n* = 222)	96	30	<0.01	193	87	<0.01	172	78	0.07
SNNP (*n* = 5613)	1030	18	0.25	4952	88	<0.01	4545	81	<0.01
Gambella (*n* = 123)	27	22	<0.01	93	75	0.07	72	58	<0.01
Harari (*n* = 81)	37	45	<0.01	40	49	<0.01	36	44	<0.01
AddisAbaba (*n* = 761)	493	39	<0.01	17	2.2	<0.01	32	44	<0.01
Dire Dawa (*n* = 151)	74	49	<0.01	39	26	*P* < 0.01	53	35	<0.01

^a^percentages indicate proportion of households in different regions, who had high HDDS, engage in farming and own livestock

The consumption of each food group was further tested against the households socio-economic score (Table 3). The analysis revealed a significant difference in the consumption of all the food groups in the studied households by their socio-economic score.

**Table 3:** Consumption of the food groups by Socio-economic score (SES)^b^

Food groups	Socio-Economic Score						X^2a^	*p-*value
	Low SES *N* = 17,921		Medium SES *N* = 6945		High SES *N* = 3129			
	No	%	*N*	%	*N*	%		
Meat	4659	26	2560	36.9	1873	59.9	1474	*P* < 0.01
Egg	1652	9.2	1050	15.1	1160	39.1	1751	*P* < 0.01
Milk	6475	36.1	1993	28.7	1141	36.5	130	*P* < 0.01
Fish	188	1.1	68	0.9	56	1.8	15	*P* < 0.01
Vegetable	14,609	81.5	5789	83.4	2439	78.0	41	*P* < 0.01
Fruit	2529	14.1	1677	24.2	1266	40.5	1300	*P* < 0.01
Oil	12,014	67.0	6004	86.5	3080	98.5	2029	*P* < 0.01
sweets	4756	26.5	3611	52.0	2793	89.3	4942	*P* < 0.01
Legumes	13,003	72.6	5671	81.6	2640	84.4	360	*P* < 0.01
Tubers	4206	23.5	2409	34.7	1743	55.7	1426	*P* < 0.01
Cereals	17,011	94.9	6741	97.1	3127	99.9	205	*P* < 0.01

^a^chi square test for trend, ^b^percentages indicate proportion of food groups consumed in each SES category

### Composition of the food groups consumed by HDDS

In addition to reporting the food groups consumed, it is important to know which food groups were predominantly consumed at different levels of HDDS. This provides information on which food groups were eaten by those with the lowest score and which ones were added for those with the higher score. In the current study, there was a notable difference in the consumption of the food groups among the households with respect to their dietary diversity score (Table 4). Cereals (95.1%), vegetables (78.5%), legumes (71.9%) and oil (69.3%) were the food groups consumed in a greater proportion among households with low HDDS. The most frequently reported food groups by the households with higher HDDS in addition to the previous ones were sweets (84.3%), meat (70.8%), tubers (65.2%), milk (62.3%), fruits (54.4%) and egg (50.5%).

**Table 4:** Pattern of consumption of the food groups by household dietary diversity score

Low HDDS			High HDDS			*P*-value
Food groups	Frequency	%	Food groups	Frequency	%	
Cereals	5403	95.1	Cereal	22,265	99.8	*P* < 0.01
Legumes	4085	71.9	Legumes	20,659	92.6	*P* < 0.01
Vegetables	4460	78.5	Vegetables	20,926	93.8	*P* < 0.01
Oil	3938	69.3	Oil	22,154	99.3	*P* < 0.01
Fruits	602	10.6	**Fruits**	12,137	**54.4**	*P* < 0.01
Tubers	1182	20.8	**Tubers**	14,546	**65.2**	*P* < 0.01
Sweets	1619	28.5	**Sweets**	18,785	**84.2**	*P* < 0.01
Egg	2784	4.9	**Egg**	11,267	**50.5**	*P* < 0.01
Meat	1290	22.7	**Meat**	15,795	**70.8**	*P* < 0.01
Milk	1545	27.2	**Milk**	13,899	**62.3**	*P* < 0.01
Fish	6	0.1	Fish	580	2.6	*P* < 0.01

Bold: to indicate food groups consumed in greater proportion (>50%) mainly among households with higher HDDS

### Pattern of Animal Source Food consumption by region and HDDS

**Fig. 2:**
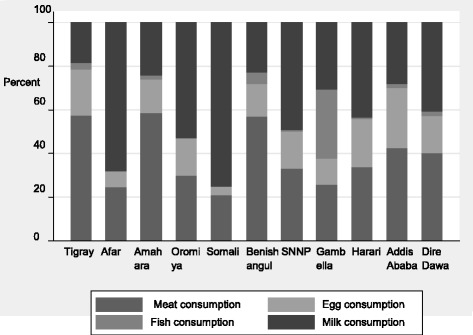
Proportion of ASF (meat, egg, fish and milk) consumption among the regions

Examining the pattern of consuming ASF showed the variability in consumption across the different regions (Fig. 2). The Somali region had the highest intake of milk where 80.7% reported milk consumption in the previous week. The lowest milk intake was found in Benishangul, with only 14.8% reporting milk consumption in the reference period. Meat was consumed in 54.3% of households in Tigray and 47.7% of households in Addis Ababa. Egg was consumed by 38.8% of the households in the capital Addis Ababa, which was a higher proportion compared to other regions. Gambella, Dire Dawa, Addis Ababa and Harari were the regions where relatively all ASF, except fish, were consumed in a better proportion compared to other regions. Fish is consumed in significant proportion (44.8%) only in households in Gambella in the reference period.

This study demonstrated that ASFs, specifically meat, egg and milk in addition to other food groups were consumed in a larger proportion (>50%) among households with higher HDDS and the variation was found to be statistically significant (Table 4).

### Predictors of HDDS

Predictors of higher HDDS were identified in this research using logistic regression analysis. Based on that, socio-economic score of the higher (AOR = 5.37, *P < 0.01*) and medium category (AOR = 1.93, *P < 0.01)*, urban residence (AOR = 1.83, *P < 0.01*), literacy (AOR = 1.79, *P <0.01*), having more than 4 household members (AOR = 1.18, *P < 0.01*) and owning livestock (AOR = 1.58, *P < 0.01)* were found to be positively associated with higher HDDS. On the other hand, the odds of having higher HDDS among female headed households was lower than that of male headed households by 11% (AOR = 0.89, *P < 0.05*). The likelihood of having higher HDDS among those households who experienced food shortage in the previous 12 months was 62% lower when compared to those households with no shock experience (AOR = 0.38, *P < 0.01*) (Table 5).

**Table 5:** Multivariate logistic regression analysis: predicting the likelihood of having high HDDS in Ethiopia

Predicting variables		Number	β	AOR (95% CI)	*P*
Sex of the household head	Male	20,921		1	
	Female	7074	−0.11	0.89 (0.80,0 .99)	0.04*
Residence	Urban	6035	0.61	1.83 (1.61, 2.08)	0.01*
	Rural	21,958		1	
Literacy status of the household head	Read and write	11,125	0.58	1.79 (1.62, 1.98)	0.01*
	Not read and write	16,871		1	
Experienced Shock in the past 12 months	Yes	7340	−0.07	0.93 (0.83, 1.03)	*0.20*
	No	20,655		1	
Illness in the past 2 weeks	Yes	2778	0.06	0.93 (0.79, 1.12)	*0.52*
	No	25,817		1	
Own livestock	Yes	20,970	0.46	1.58 (1.37–1.82)	0.01*
	No	7025		1	
Engaged in farming	Yes	21,724	0.09	1.10 (0.94–1.28)	*0.22*
	No	6271			
Food shortage in the past 12 months	Yes	4047	−0.96	0.38 (0.33–043)	0.01*
	No	23,948		1	
Household size	≤4	13,375		1	
	>4	14,620	0.16	1.18 (1.06, 1.29)	0.02*
Socio Economic Score	Low	17,921		1	
	Medium	6945	0.66	1.93 (1.71, 2.19)	0.01*
	High	3129	1.68	5.37 (4.56, 6.32)	0.01*
Age of the household head	≤20	662	0.05	1.05 (0.78,1.39)	*0.75*
	21–30	6316	0.24	1.27 (1.12, 1.44)	0.01*
	31–40	7245	0.11	1.11 (0.98 , 1.25)	*0.08*
	41–50	5411	0.04	1.04 (0.91, 1.18)	*0.54*
	>50	8359		1	

Parameter estimates were adjusted for the tabulated variables. *significant predictors at *p* < 0.05

## Discussion

This study showed that the diet of many of the households was composed of cereals, oil, legumes and vegetables. Majority of the households had low HDDS and foods from animal sources were a rare component in the household’s diets, particularly in households with low HDDS. The study also revealed that various intermediate factors like sex of the household head, socio-economic score, residence, household size and experience of food shortage and owning livestock to influence consumption of diversified diet in the households.

### Food consumption

Similar to many LMICs [34], cereals were consumed among many of the households whereas; fruits and animal source products were less consumed. The study revealed that most of the households had low HDDS, though there were notable variations across regions. It was in the two city administrations (Dire Dawa and Addis Ababa) and Harari region that a sizable proportion of households had high HDDS and also consumed ASFs in better proportion compared to other regions. Households in these regions were urban residents with higher access to markets that can supply more food varieties which may have contributed to the higher score. The growing demand by the middle class of the population and other external economic changes are causing domestic prices of food items to rise [35]. As a result, farmers are selling majority of their products to the urban consumers and many of the products are not accessible at an affordable price to the poor majority in rural areas. Moreover, urban households are believed to be exposed to information about the health benefits of a diversified diet through different media outlets [30]. Afar, Amhara and Somali were the regions where more than 80% of the households were having a low HDDS. Majority of the households in these regions are pastoralists and traditional farmers who are dependent on marginal resources whose availability are dependent on seasonality. They face challenges like recurrent droughts, flood, erratic rain fall patterns and high temperature [36, 37] that threaten their livelihood practices. Their frequent mobility as a result of the aforementioned disasters and a very traditional way of farming may have resulted in lesser productivity and then consumption of a less diversified diet.

The current study demonstrated that the proportion of ASF consumption is higher among households with higher HDDS. The multiple logistic regressions also revealed a positive and significant association between owning livestock and higher HDDS. Therefore, the increased ASF consumption could be from a direct consumption of what they own or through increased purchasing ability of the households as they generate income from owning livestock. For majority of households, livestock is an important asset of regular income that the households rely on and be disposed of in hard times to provide a safety net [38]. As reported previously [30] and demonstrated in the current study also, HDDS is positively associated with a higher socio-economic status of the households where by the households are able to purchase variety of foods including ASF to diversify their diet.

Though there is a significant difference in the consumption of all food groups in both categories, our study indicated that ASFs were introduced to the plates of households in a greater proportion in the higher HDDS category compared to the lower HDDS category. A diversified diet containing ASF is good source of essential micronutrients [15, 39]. Although ASF consumption contributes majorly to dietary quality, decreased access and relatively higher price hampers its consumption in most low income settings [40, 41].

Fruits are among the main food groups which contribute a significant share for under-consumed nutrients in many settings. They reduce the risk of many chronic diseases, help individuals achieve and maintain a healthy weight [42, 43]. Their intake is also commonly taken as an indicator of a healthy overall diet [44]. This study identified that, fruits were also among the food groups which were consumed in larger proportion in the higher HDDS category and the difference in the consumption was also found to be statistically significant. Evidence suggests that consumption of fruits is highly associated with household socio-economic status and urban residence in LMICs [45, 46]. Availability in the markets, availability of storage facilities, change in life style and cultural patterns could also result in a higher consumption of fruits among the households [47].

Consumption of sweet foods and beverages has long been in scrutiny for its risk related to obesity and chronic diseases worldwide [48]. Despite this fact, its consumption in the current study was found to be higher among households with high HDDS. The need for convenience leading to the purchase of more processed foods, availability in the urban markets and recent shifts in the pattern of dietary consumption due to increased income and urbanization [49, 50] could have resulted in its increased consumption.

The consumption of tubers and oils were also much higher in the higher HDDS households in the current sample. If one has to mention largely consumed food groups in Ethiopia, edible oil or cooking oil would be one on top. Though edible oil is one of the main consumer goods with increased price and exist in short supply, the urban consumer, where majority of households with higher HDDS live, are dependent on it for most domestic food preparations. Recent report on food production and consumption patterns in sub Saharan Africa [51] indicated that, there has been a positive growth rate in the production and consumption of cereal, oil seeds and tubers. The report also informed that there is a shift in the production of cash crops to food crops due to increase in the incident of drought conditions. This could apparently result in the increased consumption of these food groups.

### Predictors of Household Dietary Diversity Score

The current study identified that households from urban areas and high socio-economic groups have a more varied diet. This finding is comparable with a previous study in Ethiopia and central Mozambique [30, 52], which indicated that urban residence and high household income tertiles were positively associated with having high HDDS. This suggests that in the face of increasing food prices, higher income of the household enhances the capacity to purchase various food items thereby they will have access to consume a diversified and quality diet [53–55].

Experiencing food shortage in the past 12 months was negatively associated with higher HDDS. Food constraints, which can be expressed through having poor dietary practices, can occur when households experience different types of shocks. The poor dietary practices emanate from behavioral adaptations in response to food shortages in the household [56, 57] which ultimately result in a trend of consuming less diversified diet in the households. Our findings have implications for the current drought situation in Ethiopia where the livelihood of more than 10 million people is already in crisis [58]. In the face of scarce food supplies, households opt to coping strategies expressed by reduced number or size of meal which will negatively affect their nutrient intake [56, 59–61].

It is also noted in this study that male-headed households had a higher HDDS. As is common in many LMICs, men are mostly household’s decision makers and the sole source of income [62, 63] which could directly affect the variety of diet consumed in the household. . But it is also important to note that there are studies [64] that identified that female-headed households spent more on higher-quality, more expensive and protein-rich foods. This implies the need to empower [65] women through involving them in household decision making, having their own income or managing the family budget could have also a positive impact in consuming quality diet in addition to its diversity. Literacy of household heads was also positively associated with an increased HDDS. Comparable findings from other studies noted that literate household heads spent a significant amount of their food budget on nutritionally important food groups, because of a better understanding on their health benefits [66–69].

Households with more than four family members were found to be positively and significantly associated with higher HDDS. In these households, 45.3% of the household members were between 15 and 50 years of age. A finding from the 1996 Tanzanian health and demographic survey corroborated that, in a labor intensive socio-economy, households with a larger labor force could be less poor[70]. Families with a larger labor force can also invest more in agricultural practices resulting in an increase production, or increase their income by paid labor.

This analysis was based on very large data set which represents all regions of the country, and provides a good source of information for benchmarking strategies focusing on nutrition surveillance in the country. As there is within household variability of dietary intake within the days of the week, the study used a reference period of 1 week to define consumer of a food group. The dataset used does not provide information on the quantity of food consumed and the adequacy of nutrients (compared to requirements). Thus, we were unable to investigate if HDDS is associated with adequate nutrient intake. Given the fact that most people share food from a common bowl, it is difficult to quantify individual consumption and attribute the nutrient intakes [6, 71]. Nevertheless, dietary diversity score has been validated as a useful proxy indicator to assess the likelihood of meeting micronutrient requirements [71–73].

In Ethiopia many programs such as Social Safety Net, Agriculture Development Program (AGP) and Livestock Market Development [74] aim to increase household income, agricultural productivity, improve quality and diversity of household diet and as such nutritional status. These programs are believed to help realize the Sustainable Development Goals in nutrition [75]. For these programs to be effective, identifying target populations and vulnerable areas is a key. Our analysis shows that HDDS can be used to either target or evaluate the effect of the strategies employed by the above mentioned programs.

## Conclusions

Due to changes associated with urbanization, increasing income and market liberalization in many LMICs, food systems are changing, resulting in greater availability and diversity of food, although access to this food is by no means universal. So far, general trends in food consumption patterns have been documented. But, understanding of which foods are consumed by which consumer groups, where and why is pressing to know, not only for its importance for food and agricultural planning, but also for designing of interventions of preventive measures. The current study highlights the pattern of consumption of various food groups to be different across the regions, SES and the composition of food groups consumed to vary by HDDS. It also demonstrated that higher HDDS was identified among households with higher socio economic status, literate, larger and male-headed households, living in urban areas. In the light of these findings, we need to focus on nutrition sensitive interventions addressing the problem through economic and educational empowerment and modern technologies supporting agricultural practices to increase both local production and increased consumption of diversified and quality diet.

## Abbreviations

ASF: Animal Source Food
DDS: Dietary Diversity Score
HDDS: Household Dietary Diversity Score
LMICs: Low and middle income countries
SES: Socio Economic Score
SPSS: Statistical Package for Social Sciences
